# Assessment of nutritional status, physical fitness and physical activity of school going adolescents (12–15 years) in Delhi

**DOI:** 10.1186/s12887-024-04733-y

**Published:** 2024-05-14

**Authors:** Shanza Ferozi, Anu Gupta Taneja, Neha Bakshi

**Affiliations:** Department of Food and Nutrition, Lady Irwin College, University of Delhi, New Delhi, India

**Keywords:** Adolescents, Physical fitness, Nutrition status, Physical activity, Obesity

## Abstract

**Background:**

Adolescence is a distinct period that is crucial for setting the foundation for long-term health.

**Objective:**

To assess the nutritional status, physical fitness, and physical activity of adolescents.

**Methods:**

The present cross-sectional study recruited 100 adolescents purposively. Information regarding general profile and lifestyle-related factors was collected using a questionnaire. Anthropometric data such as height, weight, BMI, and body fat% were collected using appropriate equipment. Physical fitness was assessed using a battery adapted from FITNESSGRAM® and PAQ-A assessed the physical activity. Dietary intake was analysed using a 2-day 24-hour dietary recall.

**Result:**

The study revealed, 19% of the participants were overweight and 6% were obese. The majority (74%) were physically inactive and 15% had high body fat %. There was lower consumption of energy, carbohydrates, iron, and calcium, than the recommendations. Also, physical activity scores were negatively associated with macronutrient intake and trunk lift (strength and flexibility) [*p* < 0.05]. Data showed lower physical fitness scores. BMI and hand-grip strength was positively correlated [*p* < 0.05]. Push Ups (endurance) and Standing Broad Jump (power) showed a negative correlation with body fat%. Tennis ball throw and PACER (cardiorespiratory fitness) were positively associated with protein intake. A multiple regression analysis significantly showed that a unit increase in cell phone usage increases body fat% by 11.64 units. Standing broad jump increases by 38.6 cm and decreases with 28.76 cm with a unit increase in playing outside and tuitions timings respectively.

**Conclusion:**

Poor nutritional status, physical fitness, and physical activity were reported among adolescents. It is imperative to plan intervention strategies to improve the overall health of adolescents.

## Introduction

Adolescence (aged 10–19 years) is an important phase in a child’s growth with rapid physical, psychological, and cognitive development [1]. There are 1.3 billion adolescents in the world today, more than ever before, making up 16% of the world’s population [2].

In India, there are 253 million adolescents between 10 and 19 years. This age group requires good nutrition, learning, knowledge, counselling, and direction to ensure their development into healthy adults [3]. Considering this important phase of growth and development, malnutrition either under or over-nutrition is a major reason for concern among adolescents. The global prevalence of underweight among children and adolescents is 8.4% for girls and 12.4% for boys. Emerging evidence suggests that over-nutrition is also a growing population health concern among adolescents in low- and middle-income countries [4]. According to CNNS 2016-18 data 24% of adolescents were thin for their age (BMI for age <-2 SD), and 5% of adolescents were overweight or obese (BMI for age > + 1 SD). CNNS 2016-18 data also suggested that overall, food consumption patterns were similar between boys and girls which showed imbalanced dietary intake [5]. The short-term complications of undernutrition (thinness or stunting) are poor performance at school and risk of frequent infections. In the long term, under-nutrition among adolescents is associated with poor general health, and less economic productivity [6, 7]. On the other hand, over-nutrition contributes to the early development of non-communicable diseases such as diabetes, hypertension, coronary heart diseases, sleep apnoea, and cancer [8].

Physical fitness and physical activity are two important factors that affect health besides dietary intake. While physical activity is considered a behaviour with a degree of choice on behalf of the individual involved, physical fitness is an attribute, and fitness components include cardio-respiratory endurance, muscle strength, flexibility, and body composition [9, 10]. Despite all the health benefits of physical activity, most adolescents worldwide are physically inactive. It is estimated that 77.6% of boys and 84.7% of girls aged 11 to 17 years are physically inactive [11]. The modern era has brought changes in ways of life and work that are associated with lower levels of physical activity [12]. Also, decline in physical fitness are often recorded [13], which are likely influenced by the decreasing trend in physical activity and changes in body composition [14, 15].

The capacity for physical activity is intimately linked to the multi-component concept of physical fitness [16, 17]. Due to the favourable effects of high levels of fitness during childhood and adolescence on adult health, it is a significant health marker [18, 19]. Higher levels of physical fitness also reduce the chance of health issues and allow involvement in a wider range of physical activities [20, 21].

Changes in physical fitness, physical inactivity and poor nutrition status can lead to health problems later in life, such as obesity, diabetes, osteoporosis, back pain, cardiovascular disease, and cancer [22].

Studies on Indian children and adolescents have shown an association between diet patterns, physical activity level and overweight/obesity [23–25]. Considering this crucial stage of adolescence which lays the foundation for future health status and wellbeing, it is imperative to assess their physical fitness levels along with nutrition status and physical activity. Assessment of physical fitness ensures body’s ability to perform activities required for healthy living. Physical fitness can be achieved through adequate nutrition, appropriate physical activity, and adequate rest. Furthermore, there is limited research work to comprehend the relationship between different components of physical fitness with nutrition status in the Indian adolescent population. Hence, the present study aimed to assess the physical fitness, nutritional status, and physical activity of school-going adolescents (12–15 years).

## Methods

This cross-sectional study recruited 100 adolescents in the age group of 12–15 years from a government and an aided school in North Delhi. Before conducting the research, ethical clearance was taken from the Lady Irwin College Institutional Ethical Committee. Informed Consent and Assent were also taken from the parents and children respectively after providing the required information about the study. Students with any kind of disability, with chronic diseases which have an impact on physical activity and fitness like diabetes, asthma etc. and adolescents regularly involved in any sports activities were excluded from the study to maintain sample homogeneity.

General information regarding basic subject profile like, educational qualification, family income, socio-economic status, number of family members, etc. was gathered using a questionnaire. An e-questionnaire was made for data collection. The questions were asked from the participants and filled in by the researcher.

Nutritional assessment was done using anthropometric measures like weight, height, BMI, and body fat % was assessed using Tanita body composition analyser. The height and weight of adolescents were taken using valid and reliable tools. BMI was computed using Quetlet’s Index and the adolescents were classified into different categories of nutritional status using WHO 2007 growth charts [26] and IAP 2015 revised growth charts [27]. WHO Anthro plus Software was used to categorise adolescents according to BMI for age standards by WHO. For classification of nutrition status according to IAP standards, the 75th percentile and 95th percentile were characterized as overweight and obese respectively. The 3rd percentile was used to define thinness. Body fat % of adolescents was classified using standards given by Khadgawat et al., (2013) [28]. The percentage of body fat < 85th centile was considered as having normal body fat, those with a percentage of body fat between 85-95th centile as having moderate body fat, and individuals > 95th centile were considered as having elevated body fat.

The researcher collected information from the respondents on the nature and quantities of food consumed over the past 24 h. The nutritional requirements increase as the child grows and some of the nutrients in which a surge can be seen are energy, protein, calcium, magnesium, folate, iron, vitamin A, etc [29]. Two days 24-hour recalls were recorded, and the percent adequacy was calculated for energy, protein, fat, carbohydrate, iron, vitamin A and calcium. Then a comparison was made with ICMR, 2020 recommendations for dietary allowances [29] and was analyzed using dietary software. For comparison of food group intake by adolescents, NIN Dietary Guidelines for Indians (2011) were used [30]. Physical Activity was assessed using the Physical Activity Questionnaire – Adolescents (PAQ-A), which is a validated tool to assess general levels of physical activity for the last seven days. It provides a summary of physical activity score derived from eight items, each scored on a 5-point scale [31]. In the original version of this questionnaire, the first question listed 23 different physical activities. Since many of them weren’t applicable in Indian contexts, the questionnaire was changed, and 13 applicable activities were eventually retained in the final version. A score of 2.75 (> 60 min of moderate-vigorous physical activity per day) was used to detect adolescents performing enough physical activity [32].

In the study, the test battery that was used for assessing the physical fitness components like cardiorespiratory fitness, muscular strength, endurance, flexibility, and power consisted of 8 test items. PACER was used for assessing cardiorespiratory fitness, Curl Up for abdominal strength and endurance, Trunk Lift for trunk extensor strength and flexibility, Push Up for upper body endurance, Back Saver Sit and Reach for flexibility, Tennis Ball Throw for upper body power, Standing Broad Jump for lower body power, and Hand Grip Strength for maximum isometric strength. The 8 test items that are included in the test battery have appeared in Fitnessgram® 2013 [33] and are also included in various research papers [34–38]. The 8 test items included in the test battery are given in following Table 1. The Fitnessgram® (2013) reference standards were used for classifying adolescent boys and girls according to their performance on different physical fitness tests. Administration of the test battery was done by the school physical education teacher while the researcher recorded the results. On a single day, two or three physical fitness tests were administered based on the schedule of the adolescents. For Trunk Lift, Tennis Ball Throw, and Standing Broad Jump better of two attempts was recorded whereas for other tests the score for a single attempt was recorded as continued performance can improve the results which can create bias.

**Table 1:** Physical fitness test battery (Fitnessgram® 2013)

Test	Description
1. PACER	Students run back and forth between two lines 20 m apart at a progressively increasing pace, until they are no longer able to maintain the pace, or they voluntarily stop running [33].
2. Curl Up	In this test the subject lies in a supine position on the mat, knees bent, feet flat on the floor, legs slightly apart, and arms straight. The measuring strip is placed under the legs in such a way that fingertips are just resting on the nearest edge of the measuring strip [33].
3. Trunk Lift	The student lies on the mat in a prone position (facedown). Toes are pointed and hands are placed under the thighs. The student lifts the upper body off the floor, in a very slow and controlled manner, to a maximum height of 12 inches [33]. Two trials were allowed.
4. 90º Push Up	The student assumes a prone position on the mat with hands placed under, fingers stretched out, legs straight and slightly apart, and toes tucked under. The student pushes up off the mat with the arms until arms are straight, keeping the legs and back straight. The student then lowers the body using the arms until the elbows bend at a 90° angle and the upper arms are parallel to the floor [33].
5. Back Saver Sit and Reach	The student sits down at the test apparatus. One leg is fully extended with the foot flat against the face of the box. The other knee is bent with the sole of the foot flat on the floor. The arms are extended forward over the measuring scale with the hands placed one on top of the other. The student reaches directly forward with both hands along the scale and holds the position for at least 1 s. After one side has been measured, the student switches the position of the legs and reaches again [33].
6. Tennis Ball Throw	Throwing a tennis ball with one hand (the child chooses which hand) as far as possible. The child stands with the contralateral foot in front of the ipsilateral foot. The test item score (better of 2 attempts) is the distance thrown (measured in meters). This test item will help us in assessing the upper body Power [35].
7. Standing Broad Jump	The child stands with his or her feet parallel and shoulder width apart behind a starting line. Upon a signal, the child swings the arms backward and forward and jumps with both feet simultaneously as far forward as possible. The test item score (better of 2 attempts) is the distance between the starting line and the landing position (measured in centimetres). This test item will help us in assessing the upper body power [35].
8. Hand Grip Strength	Hand grip strength (HGS) is used to predict health throughout an individual’s lifetime. It is one of the field tests used the most to measure maximum isometric strength of the grip strength of both hands. Hand dynamometer was used to assess the hand grip strength [38]. The strength of participants was interpreted using the norms given by Gómez-Campos, et al. (2018) [38].

### Statistical analysis

The descriptive statistics of the participants’ baseline characteristics and responses were provided as frequency and percentage for categorical variables that were presented differently for boys and girls. A bivariate analysis was performed, using the Spearman correlation coefficient (rs), Kruskal-Wallis H test value, and Pearson correlation coefficient to indicate the strength of the association between different variables; *p* values below 0.05 were considered to indicate statistical significance. Multiple regression was run with lifestyle factors that may have an impact on dependent variables such as physical fitness, physical activity, nutrition status, and dietary intake whereas lifestyle factors like gender, the total number of family members, number of earning members, educational qualification of parents, annual income of the family, participation in any sports activity in school or neighbourhood, time spent playing outside with friends, availability of personal cell phone, use of the phone for education purpose, time spent on the phone on an average in a day, time spent watching TV, additional tuitions, frequency of tuitions, helping parents at home in household and other chores, frequency of getting involved in household chores, meals consumed in a day, and teaching about benefits of participating in sports and other activities were taken as independent variables.

## Results

### General profile of the participants

The data gathered showed that 90% of the sample consisted of boys; the majority of them lived in a nuclear family (61%) and had one earning family member (82%). Only 16% of the parents were graduates and 62% of the fathers were businessmen. None of the participants had any medical condition. The majority of them were non-vegetarian (85%) and consumed 3 major meals in a day (71%). Most of the adolescents involved in the study had a screen time of 1–2 h (74%) according to IAP (2021) guidelines recommend balancing screen use with other activities such as physical activity, schooltime, and sleep [39]. Most of the adolescents (69%) claimed that they help their parents at home. 78% of the adolescents were found to spend 1–2 h playing outside.

Data revealed that 100% of participants were consuming breakfast. It was found in our study that about 62% of adolescents were getting 9–10 h of sleep (Table 2).

**Table 2:** Demographic profile

S. No.	Category	[% (n)]
1.	Gender [ Boys / Girls ]	[90 (90) / 10 (10)]
2.	Age [ 12 years/ 13 years / 14 years / 15 years ]	[ 41 (41)/ 43 (43)/ 14 (14)/ 2 (2)]
3.	Parent’s Qualification [ Illiterate / 5-12th / Graduate ]	[ 7 (7) / 77 (77) / 16 (16) ]
4.	Parent’s Occupation [ Businessman / White Collar Workers / Blue Collar Workers ]	[ 62 (62) / 16 (16) / 20 (20) ]
5.	Eating Habits [ Vegetarian / Non – Vegetarian / Ovo – Vegetarian]	[ 6 (6) / 85 (85) / 9 (9) ]
6.	Meal Pattern [ 3 Meals / 4 Meals / 5 Meals / > 6 Meals ]	[ 72 (72) / 22 (22) / 3 (3) / 3 (3) ]
7.	Breakfast Consumption [ Consume / Don’t Consume ]	[ 100 (100) / 0 (0) ]
8.	Sleep Pattern [ 9–10 h / 8–9 h / 7–8 h / 6–7 h / 5–6 h ]	[ 40 (40) / 22 (22) / 30 (30) / 4 (4) / 4 (4) ]
9.	Screen Time [ 1–2 h / 2–4 h / 4–6 h / > 8 h ]	[ 74 (74) / 23 (23) / 1 (1) / 2 (2) ]
10.	Daily Outdoor Activity [ 30 min / 1–2 h / 2–4 h]	[ 6 (6) / 78 (78) / 16 (16) ]

### Anthropometric information

The data showed that overweight (19%) and obesity (6%) were prevalent among adolescents according to IAP 2015 growth charts, and according to WHO 2007 growth charts only 2% of adolescents were found to be severely wasted, 4% were found to be wasted, 78% had normal BMI, 10% were overweight and 2% were obese. According to body fat % cut-offs by Khadgawat et al., (2013) [28] 85% of adolescents were found to be normal while 15% were found to be moderately fat (Table 3).

**Table 3:** Distribution of adolescents according to BMI (IAP-WHO) & body fat%

BMI Category (IAP)	Category of Body Fat %			BMI (IAP)
	Normal [N (%)]	Overweight [N (%)]	Obese [N (%)]	N (%)
Thin	5 (5)	0 (0)	0 (0)	**5 (5)**
Normal	68 (68)	2 (2)	0 (0)	**70 (70)**
Overweight	9 (9)	10 (10)	0 (0)	**19 (19)**
Obese	3 (3)	3 (3)	0 (0)	**6 (6)**
**Total**	**85 (85)**	**15**	**0 (0)**	**100 (100)**
**BMI Category (WHO)**				
Severely Wasted	4 (4)	0 (0)	0 (0)	**4 (4)**
Wasted	6 (6)	0 (0)	0 (0)	**6 (6)**
Normal	70 (70)	8 (8)	0 (0)	**78 (78)**
Overweight	5 (5)	5 (5)	0 (0)	**10 (10)**
Obese	0 (0)	2 (2)	0 (0)	**2 (2)**
**Total**	**85 (85)**	**15** (15)	**0 (0)**	**100 (100)**

Table 3 demonstrates how body fat percentage classified adolescents as normal, who were overweight (11%), obese (3%), and thin (5%) according to their BMI (IAP,2015). Only 8% of adolescents with overweight BMIs were classified as overweight by body fat % cut-offs. Furthermore, 2% of normal and 3% of obese adolescents according to BMI were also classified as overweight by body fat %. A similar trend was observed in BMI classification by WHO, 2007 and body fat %. Thus, it can be said that BMI accompanied by body fat % gives a better picture of the adiposity among the sample.

### Dietary intake

**Fig. 1:**
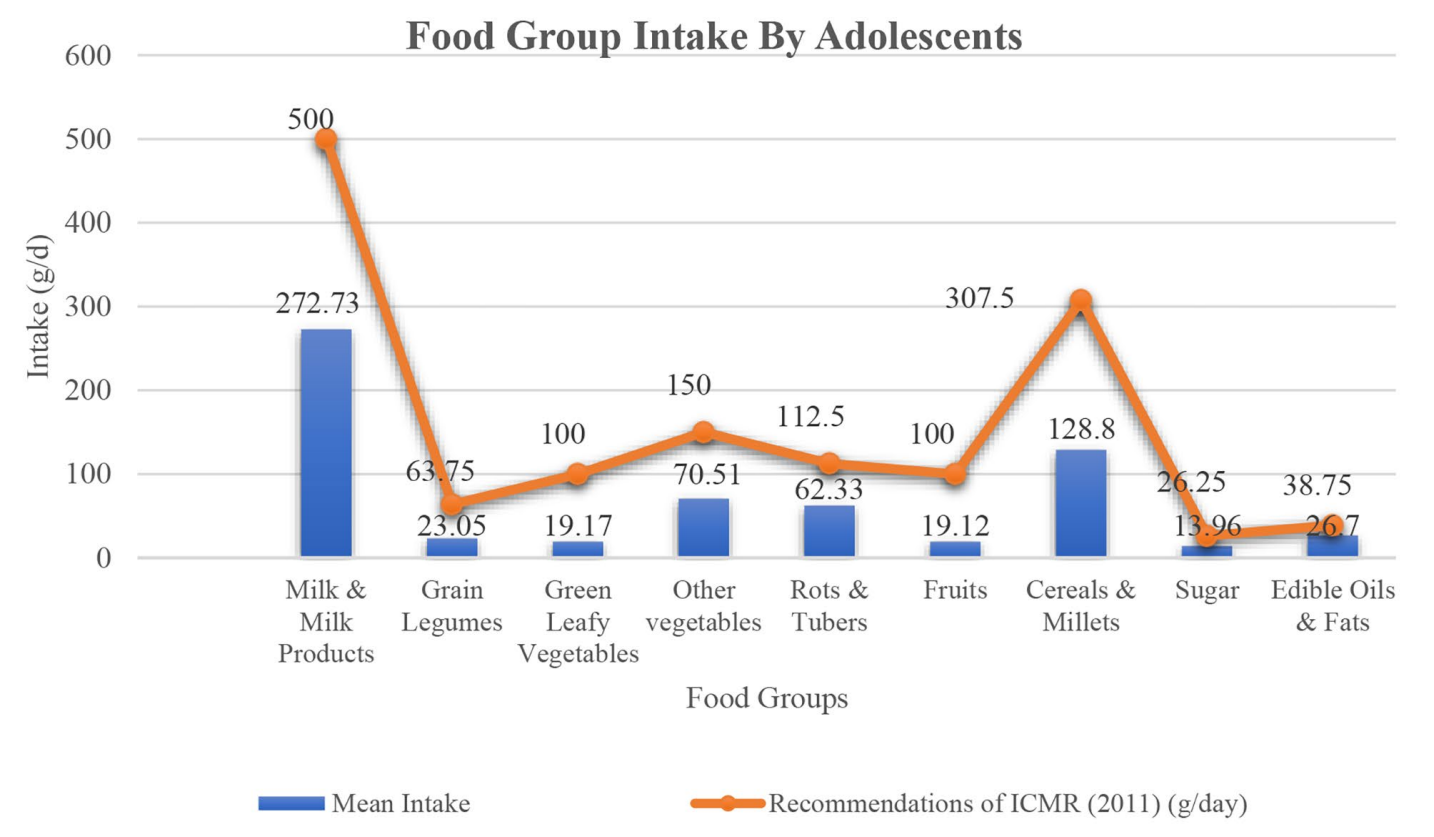
Food group intake by adolescents

**Fig. 2:**
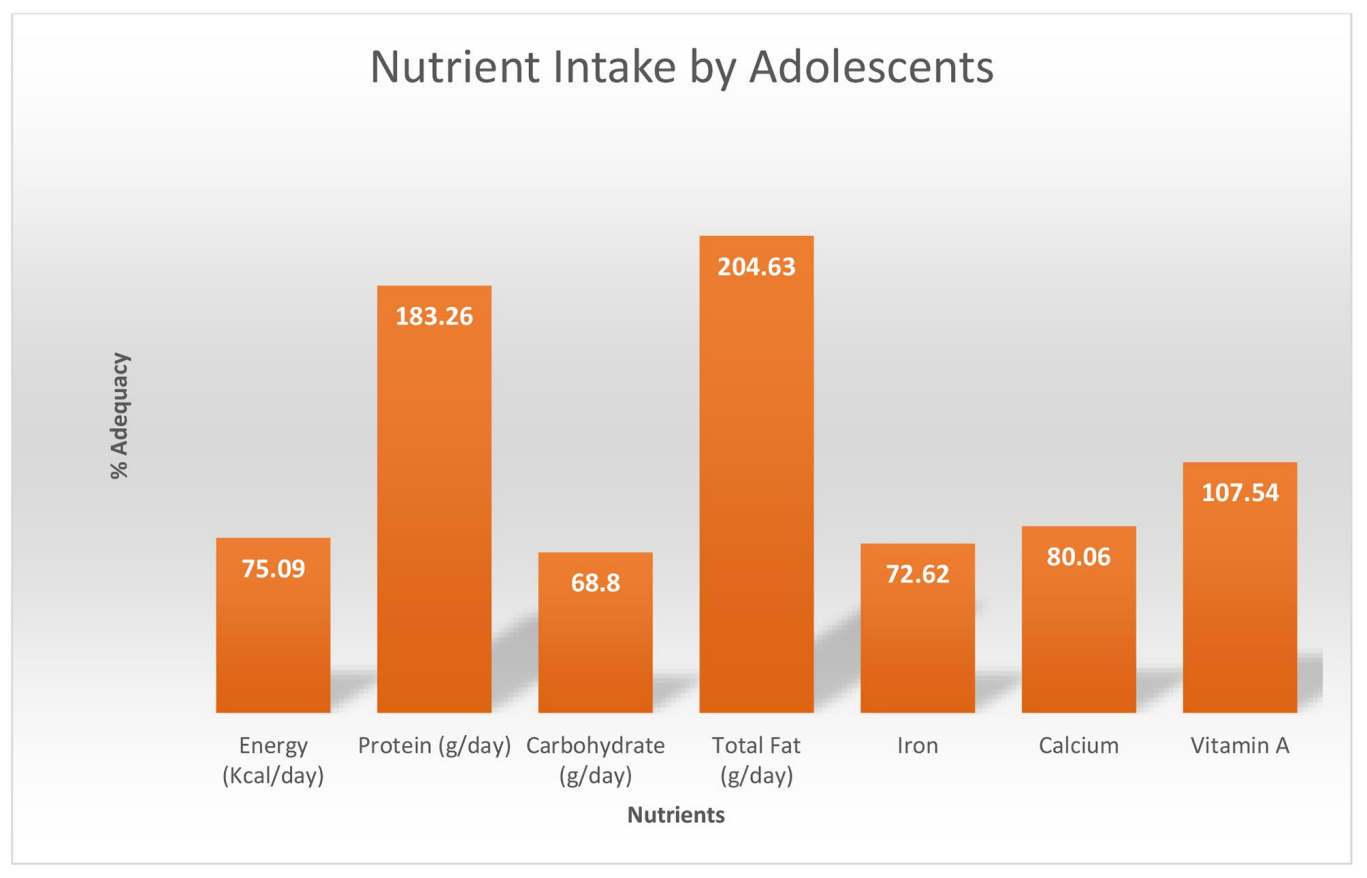
Nutrient intake by the adolescents

i. **Food Group Intake**: Fig. 1 showed that adolescents had lower consumption of milk and milk products, green leafy vegetables, other vegetables, roots and tubers, fruits, cereals, millets, and fats and oils as compared to the values given in NIN Dietary Guidelines (2011) [30].

ii. **Nutrient Intake**: The participants’ nutrient intake was calculated for vital nutrients such as proteins, iron, calcium, vitamin A, carbohydrates, fats, and energy using dietcal software. The percent adequacy was calculated and compared to the estimated average requirements for Indian adolescents by the ICMR-NIN expert committee [29]. Figure 2 shows that the percent adequacy of the nutrients specified is above 50% of the requirements. However, the intake of energy, carbohydrates, iron, and calcium was lower than the recommendations.

### Physical activity

The data gathered regarding physical activity among adolescents using PAQ-A showed that only 26% of adolescents were sufficiently active, i.e., they got a score of 2.75 which means that they were performing > 60 min of moderate-vigorous physical activity per day [32]. WHO (2020) guidelines recommend an average of 60 min per day of moderate to vigorous-intensity physical activity. Furthermore, it was found that physical activity showed a significant and negative correlation with energy (*R* (98) = -0.206, *p* = 0.04), protein (*R* (98) = -0.2, *p* = 0.045), and fat (*r* (98) = -0.26, *p* = 0.009) intake indicating that an increase in these nutrients may have a negative impact on physical activity.

### Physical fitness

**Fig. 3:**
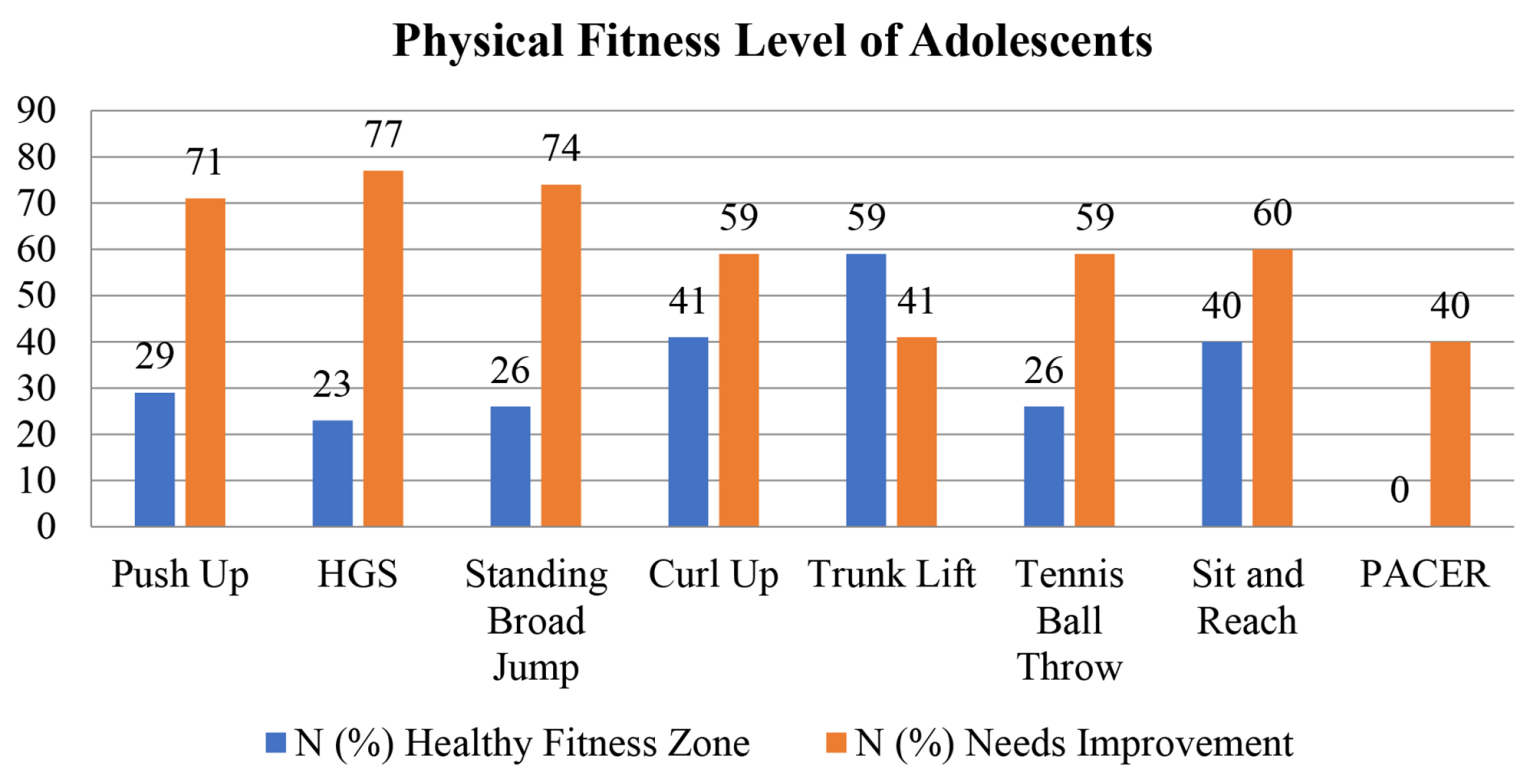
Physical fitness level of adolescents

The data gathered regarding the physical fitness of adolescents using physical fitness battery showed lower scores in different fitness tests measuring cardio-respiratory fitness (PACER), abdominal strength and endurance (Curl Up), trunk extensor strength and flexibility (Trunk Lift), upper body endurance (Push Up), flexibility (Back Saver Sit and Reach), upper body power (Tennis Ball Throw), lower body power (Standing Broad Jump), and muscular strength (Hand Grip Strength) (Table 1). Figure 3 depicts that most of the participants need improvement in different fitness tests.

### Association of physical fitness with various parameters

The association of physical fitness with physical activity, nutrition status and nutrient intake is depicted in Table 4.

**Table 4:** Association of physical fitness with different parameters

Variable	Spearman’s Rho Correlation Coefficient	*p*-value
**Association of Physical Fitness with Physical Activity**		
TRUNK LIFT(*N* = 100)	-0.206	0.04*
PACER (Laps) (*N* = 40)	-0.096	0.555
**Association of Physical Fitness with BMI**		
HAND GRIP STRENGTH (HGS) (Kgs) (*N* = 100)	0.316	< 0.001*
**Association of Physical Fitness with Body Fat %**		
PUSH-UP (*N* = 100)	-0.326	< 0.001*
STANDING BROAD JUMP (CM) (*N* = 100)	-0.273	0.006*
SIT AND REACH(*N* = 100)	0.313	0.002*
**Association of Physical Fitness with Diet Intake**		
**Push Up (No.)**		
Total Fat [g]	-0.233	0.02*
Calcium [mgs]	0.216	0.031*
Total Saturated Fatty Acids [mgs]	-0.221	0.027*
**Curl Up (No.)**		
Energy in Kcal (ENERC) [Kcal]	0.202	0.044*
Iron [mgs]	0.265	0.008*
Total Polyunsaturated Fatty Acids [mgs]	0.223	0.025*
**Tennis Ball Throw (Meter)**		
Protein [g]	0.078	0.048*
**PACER (Laps)**		
Protein [g]	0.328	0.039*

#### Physical fitness & physical activity

A significant negative association (*R* (98) = -0.206, *p* = 0.04) of physical activity was observed with trunk lift only, which assesses trunk extensor strength and flexibility. It thus indicates that the higher the physical activity level, lower the trunk extensor strength and flexibility. The data also revealed a negative association with most of the fitness tests except for sit and reach though the results weren’t significant. Thus, it could be said that higher physical activity is not synonymous to good physical fitness (Table 4).

#### Physical fitness and BMI

A significant positive correlation (*R* (98) = 0.316, *p* = 0.001) was observed between BMI and physical fitness test that measures muscular strength, i.e., Hand Grip Strength which indicates that with an increase in BMI muscular strength increases. Correlations of BMI with physical fitness tests that measure strength, endurance, and flexibility were found positive, but they were not significant. A negative correlation was observed between BMI and physical fitness tests that measure abdominal strength (Curl Up), cardiorespiratory fitness (PACER), and lower body power (Standing Broad Jump), indicating that an increase in BMI, decreases fitness scores, but the results were not significant (Table 4).

#### Physical fitness and body fat %

A significant negative correlation was observed between body fat% and push-up (endurance) (*R* (98) = -0.326, *p* = 0.001) and Standing Broad Jump (lower body power) (*R* (98) = -0.273, *p* = 0.006) indicating that performance in fitness tests decreases as the body fat % increases. Also, a significant positive correlation is observed between body fat % and Sit and Reach (flexibility) (*R* (98) = 0.313, *p* = 0.002) which showed higher body fat% is associated with higher sit and reach scores indicating that fat% does not affect flexibility (Table 4).

#### Physical fitness and diet intake

The fitness parameters were assessed for their association with all the major and micronutrients. The data presented depicts only significant values of the association between them. Push-ups scores showed a significant negative correlation with fat (*R* (98) = -0.233, *p* = 0.02) intake and a significant positive correlation with calcium (*R* (98) = -0.216, *p* = 0.03) indicating that higher the fat intake was correlated to lower endurance level whereas for calcium intake higher intake was correlated with increased endurance levels. Curl Up scores showed a positive correlation with most of the nutrients assessed and but a significant result was observed with energy (*R* (98) = 0.202, *p* = 0.04), iron (*R* (98) = 0.265, *p* = 0.008), and PUFA (*R* (98) = 0.223, *p* = 0.025) indicating that an increase in these nutrients may increase abdominal strength and endurance. Tennis ball throw showed a significant and positive correlation with protein intake (*R* (98) = 0.078, *p* = 0.048) indicating that an increase in protein intake may increase upper body power. PACER showed a positive and significant correlation with protein intake (*R* (98) = 0.328, *p* = 0.039) indicating that an increase in protein intake may improve cardiorespiratory fitness (Table 4).

#### Physical fitness and lifestyle factors

A multiple regression analysis of various independent variables like gender, the total number of family members, number of earning members, educational qualification of parents’ etc. was run with physical fitness tests that were taken as dependent variables.

It was found that a unit increase in time spent playing outside increased the standing broad jump (lower body power) scores by 38.6 cm. Similarly, a unit increase in time spent on taking tuition in a week reduces the standing broad jump by 28.76 cm. These results were found to be statistically significant (Table 5). Through a multiple regression analysis of various lifestyle factors described above (independent variables) with body fat % (dependent variable) it was found that a unit increase in the usage of the phone may significantly increase the body fat % by 11.64% (*p* = 0.014) (Table 5).

**Table 5:** Multiple regression analysis of dependent variables: standing broad jump, body fat % and BMI with different independent variables

Variables	Coefficient	t	Significance level	R^2^	*P* value of the model
**Standing Broad Jump**					
Annual income of the Family	24.515	1.986	0.054	0.611	< 0.001*
Participation in any sports activity in school or neighborhood like playing football, basketball, kho-kho, kabaddi, dancing, etc.	19.914	1.954	0.058		
Time spent on playing outside in a day with friends	38.616	2.382	0.022*		
Time spent on taking tuition in a week	-28.756	-2.912	0.006*		
**Body Fat %**					
Usage of Phone for education purpose	11.644	2.153	0.037*	0.508	0.014*

Note: To reduce the length of the table only significant results are shown

Through a multiple regression analysis of nutrient intake (independent variable) with BMI (dependent variables) it was found that a unit increase in energy, protein, and carbohydrate intake significantly increases BMI by 0.18 kg/m^2^, 0.73 kg/m^2^, and 0.72 kg/m^2^ respectively (Table 6).

**Table 6:** Multiple regression analysis of diet intake (Independent variable) with BMI (Dependent variable)

Variables	Coefficient	t	Significance level	R^2^	*p*-value of the model
Protein [g]	0.733	2.103	0.039*	0.270	0.008*
Total Fat [g]	1.513	1.95	0.055		
Carbohydrate [g]	0.719	2.03	0.046*		
Energy in Kcal [Kcal]	0.182	2.105	0.038*		

Note: To reduce the length of the table only significant results are shown

## Discussion

Adolescence is an essential developmental stage that establishes the foundation for future health. Hence, it is crucial to keep a close watch on their growth and development. Adolescents’ overall health and development are greatly influenced by several factors, including physical fitness, nutritional consumption, and physical activity.

Indian research on children and adolescents has revealed a link between dietary habits, degree of physical activity, and overweight/obesity [23–25]. However, little research has been done to understand the association between various aspects of physical fitness and nutritional status in the teenage Indian population. Therefore, the current study’s objective is to evaluate the physical fitness, nutritional condition, and physical activity of adolescents (12–15 years old) at North Delhi Schools.

Maintaining optimum nutrition status during this phase of life might be beneficial in adult life. Obesity during childhood can harm the body in a variety of ways, now and in the future. For children and adolescents, BMI screens for potential weight and health-related issues. Adolescents were classified according to WHO 2007 growth charts [26] and IAP 2015 revised growth charts [27]. A higher proportion of adolescents were found to be overweight and obese using IAP 2015 charts (as against the WHO 2007 references) and similar results were observed by Oza et al., (2021) [41]. Body fat % categorised a higher proportion of adolescents in the normal category and less proportion of adolescents fall under the overweight-obese category when compared with BMI (Table 3). Another previous study also depicted that the prevalence of overweight and obesity was found to be less with body fat % as compared with BMI [42].

Lifestyle factors like breakfast consumption, sleep hours, screen time, etc. are some of the factors that are very important for appropriate growth and development. Adolescents generally require about 9–9.30 h of sleep per night [43]. According to IAP (2021) guidelines adolescents’ screen time should be balanced with other activities that are required for overall development [39]. Excessive screen time among school-going children has been associated with physical inactivity and poor eating behaviour which could lead to an increased risk of being overweight and obese [44]. The present study showed appropriate screen time and sleep duration among the recruited adolescents (Table 2). Maintaining appropriate nutrition status requires adequate nutrient intake, adolescence is a challenging stage when it comes to food selection and opting for healthy food items. Previous studies have shown a lower intake of proteins, fruits, and vegetables and a higher intake of high-fat salt and sugar foods (HFSS) [45, 46]. The present study also depicted a lower intake of milk and milk products and fruits and vegetables.

Physical fitness is an important component of health that can play a critical role in enhancing the overall health and fitness of an individual. It is even more important for adolescents, as this phase lays the foundation for adult life and health. Improved physical fitness has positive health benefits like improved bone health, mental health, and quality of life. It also aids in the prevention of obesity and cardiovascular disease later in life. In the present study, only a small percentage of the study sample showed a sufficient level of physical fitness. Similar results were observed in previous studies also [47, 48].

Physical activity plays a crucial role in maintaining the overall health of adolescents. Regular physical activity can help children and adolescents improve cardiorespiratory fitness, build strong bones and muscles, control weight, reduce symptoms of anxiety and depression, and reduce the risk of developing health conditions [49]. Only 26% of adolescents were found to be sufficiently active while 74% were found to be inactive. WHO Physical Activity Profile 2022 [50] also showed similar trends and found that 72% of Indian adolescent boys and 76% of girls were physically inactive. The present study showed a significant negative association between physical activity and trunk lift scores which assesses trunk extensor strength and flexibility. That is, the higher the physical activity level, the lower the trunk lift scores. The data also revealed a negative association with most of the fitness tests except sit and reach though the results weren’t significant. Thus, it could be said that it is not necessary that with increased physical activity, fitness also increases. Similar results were given by Malina (2001) suggesting that a large part of the variability (80–90%) in health-related fitness is not accounted for by physical activity [51].

Most of the adolescents who performed well in all the tests had normal BMI & body fat % and our result is in line with the previous findings [37, 51]. The present study also showed that grip strength performance which assesses muscular strength was better in adolescents with higher BMI and this result is consistent with a previously done study [52]. Push-ups (endurance) and standing broad jump (power) showed a negative significant correlation with body fat % indicating that performance in these tests decreases as the body fat % increases. It could be because with increased fat % body weight also increases and standing broad jump is affected by increased weight and our result was in line with the previous study depicting impact of body fat% on the physical fitness levels [53].

The assessment of diet in relation to physical fitness showed that Curl Up had a positive and significant relation with energy, iron, and PUFA indicating that an increase in their intake may increase abdominal strength and endurance. Also, tennis ball throw and PACER showed a significant and positive correlation with protein indicating that an increase in protein intake may increase upper body power and may improve cardiorespiratory fitness (Table 4).

Through a multiple regression analysis, it was significantly found that a unit increase in time spent playing outside increased the standing broad jump (power) scores by 38.6 cm. Similarly, a unit increase in time spent on taking tuition in a week reduces the standing broad jump by 28.76 cm (Table 5). Hence it is crucial to focus on the physical fitness of an adolescent along with academics. Analysing and interpreting physical fitness along with physical activity and nutrition status is imperative to assess the wholistic development of adolescent is the main inference of this study. Though, the study had few limitations such as the study was performed on a small sample size, hence, the results cannot be generalized for the population. Students especially girls were not willing to perform fitness tests; hence, gender differences could not be studied. PACER and Standing Broad Jump were time-consuming tests, due to time constraints they could not be performed on the entire sample size of 100 adolescents.

However, the study emphasizes that physical activity, physical fitness, dietary intake, and nutrition status are all interrelated with each other. Therefore, adolescents need to be enlightened about the importance of good dietary intake along with appropriate physical activity and fitness regimes so that they have improved overall health.

## Conclusion

Adolescence being an important phase of life needs sufficient care, good nutrition, adequate physical fitness, and physical activity, these factors play a critical role in growth and development. The present study lays the foundation for future interventional research studying the impact of physical fitness and health-related aspects among adolescents. Therefore, adolescents need to be motivated to include good nutrition and appropriate physical fitness and physical activity regimes in their daily routine.

## Data Availability

The datasets used and/or analysed during the current study available from the corresponding author on reasonable request.
